# Hyperspectral Imaging for the Nondestructive Quality Assessment of the Firmness of Nanguo Pears Under Different Freezing/Thawing Conditions

**DOI:** 10.3390/s19143124

**Published:** 2019-07-15

**Authors:** Zhe Zhang, Huiqing Shang, Huaiwen Wang, Qiumei Zhang, Susu Yu, Qiaoyan Wu, Jinjin Tian

**Affiliations:** 1 Tianjin Key Laboratory of Refrigeration Technology, Tianjin University of Commerce, Tianjin 300134, China; 2 School of Mechanical Engineering, Tianjin University of Commerce, Tianjin 300134, China

**Keywords:** hyperspectral imaging, freezing/thawing, Nanguo pear, firmness

## Abstract

Firmness changes in Nanguo pears under different freezing/thawing conditions have been characterized by hyperspectral imaging (HSI). Four different freezing/thawing conditions (the critical temperatures, numbers of cycles, holding time and cooling rates) were set in this experiment. Four different pretreatment methods were used: multivariate scattering correction (MSC), standard normal variate (SNV), Savitzky-Golay standard normal variate (S-G-SNV) and Savitzky-Golay multiplicative scattering correction (S-G-MSC). Combined with competitive adaptive reweighted sampling (CARS) to identify characteristic wavelengths, firmness prediction models of Nanguo pears under different freezing/thawing conditions were established by partial least squares (PLS) regression. The performance of the firmness model was analyzed quantitatively by the correlation coefficient (R), the root mean square error of calibration (RMSEC), the root mean square error of prediction (RMSEP) and the root mean square error of cross validation (RMSECV). The results showed that the MSC-PLS model has the highest accuracy at different cooling rates and holding times; the correlation coefficients of the calibration set (R_c_) were 0.899 and 0.927, respectively, and the correlation coefficients of the validation set (R_p_) were 0.911 and 0.948, respectively. The accuracy of the SNV-PLS model was the highest at different numbers of cycles, and the R_c_ and the R_p_ were 0.861 and 0.848, respectively. The RMSEC was 65.189, and the RMSEP was 65.404. The accuracy of the S-G-SNV-PLS model was the highest at different critical temperatures, with R_c_ and R_p_ values of 0.854 and 0.819, respectively, and RMSEC and RMSEP values of 74.567 and 79.158, respectively.

## 1. Introduction

In the process of fruit freezing and refrigeration, the existence of ice crystals will cause mechanical damage to the microstructures of fruit, such as the cell membrane and cell wall [1,2], which directly determines the fresh-keeping state during fruit storage. At present, most of the research has focused on the analysis of fruit quality during postharvest storage [3], but there is a lack of nondestructive and rapid detection methods.

Hyperspectral reflectance imaging mainly utilizes the spectral and image information acquired by light reflection after absorption by imaging through fruit and vegetable tissues [4]. Depending on the obtained high-resolution spatial information and spectral information, the characteristic wavelengths of fruits and vegetables can be extracted, and then the comprehensive quality can be quickly and effectively detected. Hyperspectral applications are mainly focused on hyperspectral remote sensing [5,6,7] and food quality assessment [8,9,10]. Hyperspectral technology has been successfully applied to meat tenderness prediction [11], microbial contamination assessment [12] and fruit and vegetable defect detection [13,14,15] and many other fields. Fan S. et al. [16] researched the quality of the prediction models of pears using hyperspectral reflectance imaging. The results show that the R and RMSEP of the model combined competitive adaptive reweighted sampling, successive projections algorithm and partial least squares (CARS-SPA-PLS) were 0.876 and 0.491, respectively. Li B. et al. [17] used visible-near infrared hyperspectral imaging technology to nondestructively identify pear varieties and predict soluble solids and found that the model that combined successive projections algorithm and partial least squares (SPA-PLS) was superior to the PLS model. Yu K.Q. et al. [18] researched the application of visible-near infrared hyperspectral imaging for the detection of defective characteristics in loquat and found that the optimal model combined competitive adaptive reweighted sampling with partial least squares discriminant analysis, namely, the CARS-PLS-DA model, and the qualitative identification between defective and normal loquat samples could be realized. Cai Z.Y. et al. [19] used visible-near infrared hyperspectral imaging technology to nondestructively test the water content of Ningxia Cabernet Sauvignon grapes and found that the partial least squares regression (PLSR) model based on the multivariate scattering correction spectrum was superior to the others, and the R and RMSEP of the validation model were 0.806 and 0.144, respectively. Nicolaï B.M. et al. [20] used near-infrared hyperspectral imaging to nondestructively test for bitter pits in apples and constructed a discriminant with a PLS calibration model. Wang Y. et al. [4] used hyperspectral imaging technology to identify maize haploid kernels to overcome the current limitations of automatic haploid identification, and a fast and accurate identification method of near-infrared hyperspectral imaging was explored. Wei Y. et al. [21] researched the moisture content of tea leaves by hyperspectral imaging, and a least-squares support-vector regression (LS-SVR) model was established to automatically test the moisture content of the harvested tea leaves, and so the purpose of identifying the fronts and backs of leaves was achieved. Tung K.C. et al. [22] used hyperspectral imaging to evaluate water stress and established a modified partial least squares regression (MPLSR) model; the correlation coefficient was 0.826, and the model also provided a strong foundation for the development of future agricultural precision irrigation systems. Huang M. et al. [23] used hyperspectral imaging to establish a PLSR model for predicting the color and moisture content of dried soybeans and found that the PLSR model performs well on moisture content (the R_p_ was 0.971, and the RMSEP was 4.7%).

Hyperspectral imaging technology combines imaging technology and spectral technology and highlights the advantages of both. The image and spectral information of fruit quality can be obtained when testing for fruit quality. Moreover, the obtained image and spectral information can be combined to better test fruit quality. Infrared imaging technology has the advantages of fast measurement, easy operation and high detection sensitivity, whereas visible imaging technology is vulnerable to the influence of external light sources and yields errors. Visible imaging technology has the limitation of long testing times and relies on surface detection. Therefore, hyperspectral imaging technology has become the optimal method for firmness testing.

The existing research on the nondestructive testing of Nanguo pears based on hyperspectral imaging is not related to firmness. The effect of temperature load on the firmness of Nanguo pears by using hyperspectral imaging are researched in this paper. The aim is to analyze the firmness change data during the freezing process of Nanguo pears to establish a quantitative relationship between the temperature load and the firmness of Nanguo pears based on hyperspectral imaging. The hyperspectral prediction models under different freezing/thawing conditions will be established, and the effect of different pretreatment methods on model accuracy will be researched.

## 2. Experimental Materials and Methods

### 2.1. Experimental Materials

In total, 300 samples of Nanguo pears from Anshan, Liaoning Province, were selected as the experimental material in this paper and were purchased from a local market in Tianjin. The material was mostly uniform, individual differences were small, the internal structure and freshness of the fruits were largely similar; the pears were similar in color with longitudinal diameters ranging from 4.7 cm to 5.2 cm, transverse diameters ranging from 5.5 cm to 5.8 cm and weights ranging from approximately 50 g to 75 g. Each group comprises 120 samples: 88 pears for calibration set modeling and 32 pears for validation set modeling. To remove the influence of time and temperature on the measurements, all spectral measurements and firmness measurements for each group of samples were completed during the same time period.

### 2.2. Experimental Equipment

A constant temperature and humidity testing machine [24] (Hung Ta Instrument Co., Ltd., −70 °C~100 °C) was used to control the temperature of the Nanguo pears.

**Figure 1:**
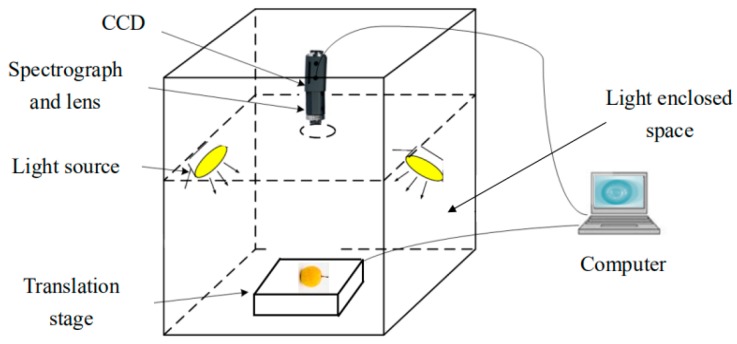
Hyperspectral imaging system.

**Figure 2:**
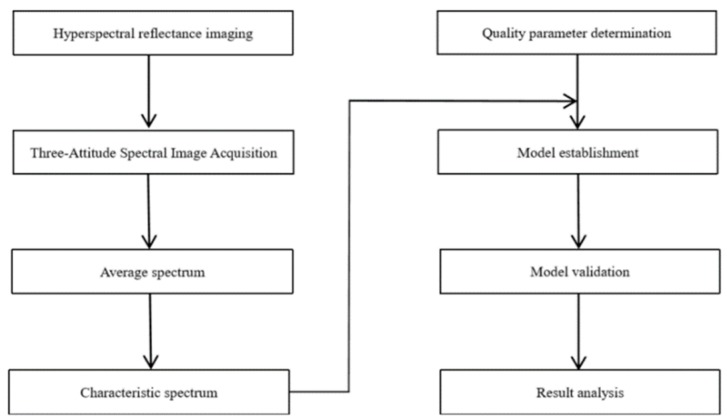
Experimental procedure.

Hyperspectral images of the Nanguo pears were acquired by using a hyperspectral imaging acquisition system (Imspector N17, Spectral Imaging Ltd., Oulu, Finland). A line-scanning CCD hyperspectral camera (Zelos-258GV, Kappa optronics GmbH, Gleichen, Germany), a translation stage (PSA200-11-X, Zolix., Ltd, Beijing, China) and illumination units (four 35 W halogen tungsten lamps, HSIA-LS-TDIF, Zolix., Ltd, Beijing, China) were included in the HSI system [25]. The wavelength range of the hyperspectral imaging spectrometer is from 900 nm to 1700 nm. In total, 256 spectral bands were recorded by the system, and the spectral resolution was approximately 3 nm. The hyperspectral schematic diagram is shown in Figure 1. The Nanguo pear is placed horizontally to obtain its circumferential equatorial plane, which contains three planes. The circumferential equatorial plane of each the two adjacent positions is 120 degrees apart, and the experimental procedure is shown in Figure 2.

### 2.3. Experimental Methods

The temperature of the Nanguo pears was controlled by a constant temperature and humidity testing machine. The first group was the fresh group, and it served as the control group. The standard experimental scheme is shown in Table 1:

In the different experiments, only the required parameters were changed, and other parameters were set to standard sample parameters. The freezing/thawing conditions at different numbers of cycles were divided into three groups (one cycle, two cycles, and three cycles), and the other freezing/thawing conditions are shown in the following tables. The experimental scheme at different cooling rates, different holding times and different critical temperatures are shown in Table 2, Table 3 and Table 4, respectively.

### 2.4. Firmness Determination

Firmness was determined by a texture analyzer (TA.XT plus, Stable Micro Systems Ltd., Godalming, UK) [26]. The firmness was extracted at a speed of 1 mm/s, and a P/2N probe (2 mm) was used in this research. The compression depth was set to 10 mm. The average force (g) from 2 to 3 s during the penetration process was calculated as the firmness (g).

To ensure the accuracy of the determination, the instrument should be calibrated before it is used. Then, the position of the Nanguo pear should be perpendicular to the probe, and the required parameters should be set during the procedure.

## 3. Acquisition and Processing of Hyperspectral Images

### 3.1. Acquisition of Hyperspectral Images

To reduce the influence of the dark currents on image quality in the acquisition system, black-and-white correction was carried out to improve the accuracy of the prediction model. Therefore, the white calibrated image (*W*), the black calibrated image (*B*) and the original hyperspectral image (*I*_0_) were measured by this experiment, and finally, the calibrated hyperspectral image (*I*) was calculated according to Equation (1):

(1) $$I=\frac{I_{0}-B}{W-B}\times 100\%$$

The hyperspectral image corrected by Equation (1) had 256 bands. Then, the minimum noise fraction transform (MNF) was introduced to denoise the image; this method can arrange the components according to the image quality [27]. Finally, the region of interest (ROI) was tailored to extract the spectral reflectance of all the pixels, and the average spectrum of each sample image was obtained.

**Figure 3:**
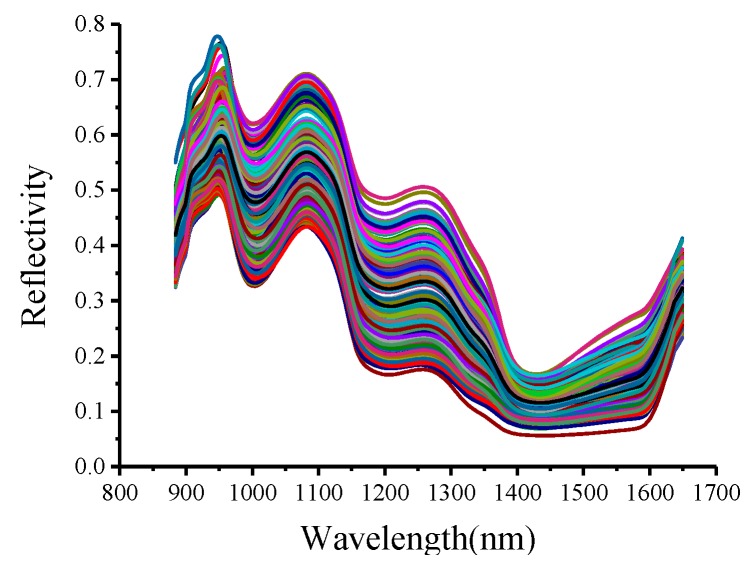
Average reflectance spectra of the 300 Nanguo pear samples.

The original reflectance spectra were extracted from all 300 samples from both the fresh and frozen groups in this experiment. The original reflectance spectra are shown in Figure 3. As shown in Figure 3, the trends in the spectral reflectance are similar. The absorption peaks of the samples are observed at 970–980 nm. In the range of 1400–1500 nm, Nanguo pears have stronger absorption than other fruits. Some changes in the spectral curves represent the characteristics of Nanguo pears, which include hidden information about different components in the pear. The differences in the spectral reflectance of the Nanguo pears are mostly concentrated at 950–1400 nm. The differences in spectral values are mainly related to the quality changes in the Nanguo pear: the change in the physical and chemical properties of Nanguo pear under different freezing conditions.

Hyperspectral imaging can receive the spectral reflectance signal, mainly those pixel points of the part needed to test the pear, and can obtain the corresponding characteristic wavelength of the pear parameters. The main components (such as the water content and the sugar content) of the pear can be effectively evaluated by spectral reflectance, and they are closely related; the water content is related to the firmness of the pear.

### 3.2. Pretreatment of the Hyperspectral Images

The obtained hyperspectral data in the range of 950–1400 nm were imported into the analysis software Unscrambler 9.7 for pretreatment. The spectral reflectance values were different and reflected the quality of the materials after different pretreatments.

**Figure 4:**
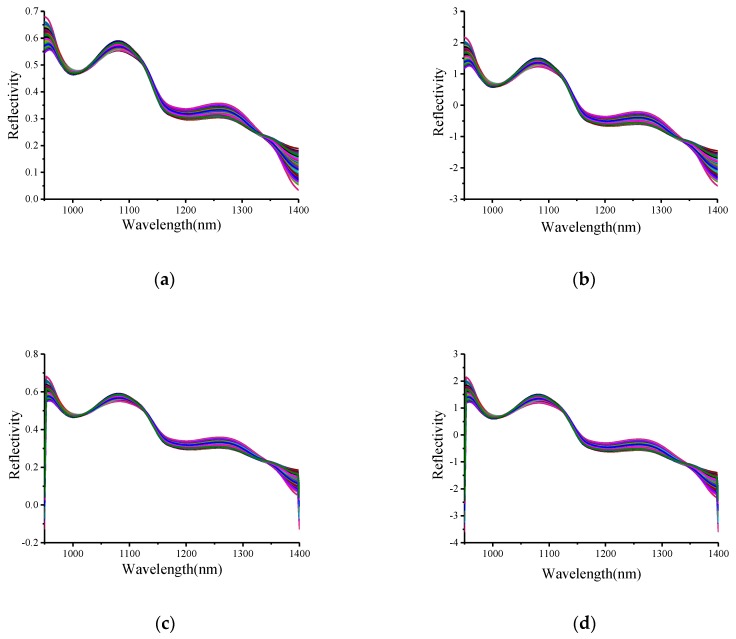
The hyperspectral curves of different pretreatment methods processed for different numbers of cycles: (**a**) MSC; (**b**) SNV; (**c**) S-G-MSC; and (**d**) S-G-SNV.

Four pretreatment methods were used in this research: MSC [28], a data processing method that can correct the scattering of each spectrum, obtain a relatively ideal spectra, effectively eliminate the impact of scattering and improve the absorption information of related spectra; SNV [29], or standard normalization, which is similar to MSC and is used to process each original spectral dataset (with this method, the error caused by scattering between samples can be eliminated); S-G-SNV and S-G-MSC, namely, the S-G convolution smoothing methods [30], are used on the basis of SNV and MSC, respectively, and then the data obtained are fitted by the least squares method. Take the freezing treatment conditions with different numbers of cycles as an example. The spectral pretreatment results are shown in Figure 4.

To solve the problem of poor prediction accuracy in the full spectrum model, the data size was reduced by precisely measuring several key wavelengths. Thus, this method can quickly and accurately analyze pear quality; quantitatively or qualitatively [31]. Furthermore, the selection of key variables reduced the computational time and makes the model meet the requirements of online applications [32].

**Figure 5:**
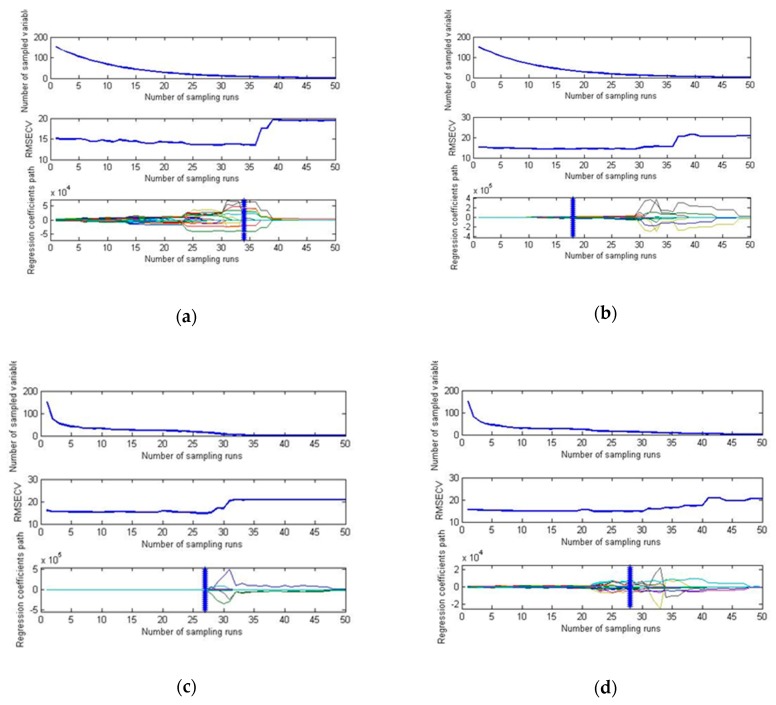
Hyperspectral characteristic wavelength selection process based on the CARS algorithm pretreated by (**a**) MSC; (**b**) S-G-MSC; (**c**) S-G-SNV; and (**d**) SNV.

CARS was proposed by Li et al. [33] in 2009. It is an innovative and useful variable selection algorithm. The characteristic wavelength was extracted by CARS. Hyperspectral data in the range of 950 nm-1400 nm were read into MATLAB. The data were pretreated by MSC, S-G-MSC, S-G-SNV and SNV. The distributions of the root mean square error are obtained by cross validation, as shown in Figure 5.

From Figure 5, it can be found that the number and the eigenvectors of the characteristic wavelengths extracted by the CARS algorithm after different pretreatments are different, and this difference has an impact on the quality of the model. The values of the characteristic wavelengths under different numbers of freezing treatment cycles are shown in Table 5.

## 4. PLS Modeling

The PLS algorithm mainly combines factor and regression analysis. Therefore, the main calculation equations [34] are as follows:

(2) X=TP+E

(3) Y=UQ+F

(4) U=TL

*T* is the score matrix of the spectral matrix (*X*), *P* is the load matrix of *X*, *U* is the score matrix of the quality attributes matrix (*Y*), *Q* is the load matrix of *Y*, *E* and *F* are error matrices when the model is fitted by *X* and *Y*, and *L* is the regression coefficient matrix.

Finally, the equation for predicting unknowns is obtained, and *Y_un_* is calculated from this equation:

(5) $$Y_{\mathrm{un}}=T_{\mathrm{un}}\mathrm{LQ}+F$$

In this research, the PLS method was used to establish a calibration model between the hyperspectral data of Nanguo pears and their quality attribute (firmness). The accuracy of the model is evaluated by R_c_, RMSEC, R_p_ and RMSEP. The equations are as follows.

(6) $$R=\frac{\sum_{i=1}^{I_{c}}{\left(\hat{y_{i}}-y_{i}\right)}^{2}}{\sum_{i=1}^{I_{c}}{\left(\hat{y_{i}}-y_{m}\right)}^{2}}$$

(7) $$RMSEC=\sqrt{\frac{1}{I_{c}-1}\overset{I_{c}}{\underset{i=1}{\sum }}{\left(\hat{y_{i}}-y_{i}\right)}^{2}}$$

(8) $$RMSEP/RMSECV=\sqrt{\frac{1}{I_{p}-1}\overset{I_{p}}{\underset{i=1}{\sum }}{\left(\hat{y_{i}}-y_{i}-bias\right)}^{2}}$$

(9) $$bias=\frac{1}{I_{p}-1}\overset{I_{p}}{\underset{i=1}{\sum }}\left(\hat{y_{i}}-y_{i}\right)$$

where *I_c_* is the number of corrected samples, *I_p_* is the number of predicted samples, *y_i_* is the true value of the *i*-th sample, $\hat{y_{i}}$ is the predicted value of the *i*-th sample, and *y_m_* is the mean of the measured values for all the samples.

## 5. Results and Analysis

The statistical data of the firmness values of the 300 Nanguo pears are shown in Table 6.

### 5.1. Firmness Prediction Model for Different Numbers of Cycles

To analyze the differences in the detection effect from the models with different spectral pretreatment methods, four pretreatment methods (MSC, S-G-MSC, SNV, S-G-SNV) were used to pretreat the hyperspectral Nanguo pear data. In total, 88 Nanguo pear samples were used as calibration set samples. The samples were numbers 1 to 22 from the fresh group, numbers 31 to 52 from the one cycle group, numbers 61 to 82 from the two cycles group and numbers 91 to 112 from the three cycles group. In total, 32 Nanguo pear samples, which were numbers 23 to 30 from the fresh group, numbers 53 to 60 from the one cycle group, numbers 83 to 90 from the two cycles group and numbers 113 to 120 from the three cycles group, were used as validation set samples to carry out the modeling and comparative analysis.

**Figure 6:**
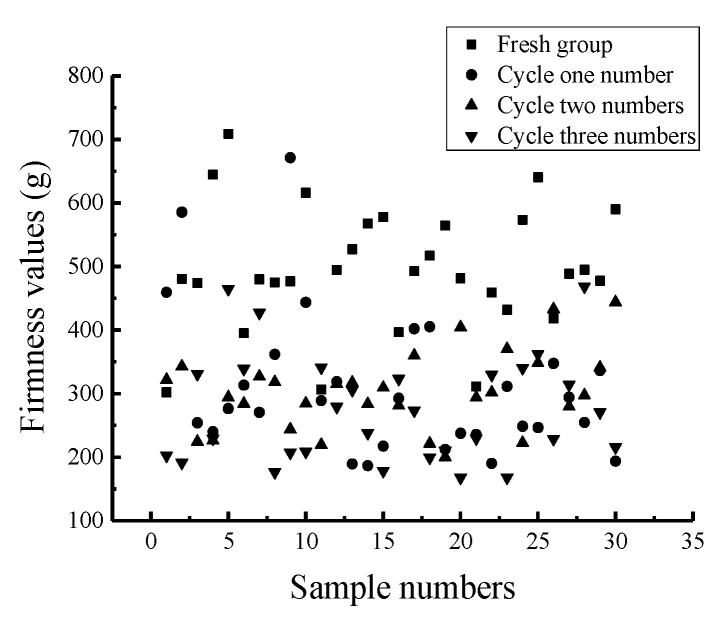
Scatter plot of the distribution of the firmness values of the Nanguo pears.

The experimental scheme is shown in Table 1. The statistical analysis results of the firmness values of the 120 Nanguo pears treated with different freezing cycles are shown in Table 7. The scatter plot of the firmness distribution is shown in Figure 6.

From Table 7, it can be concluded that the firmness value of the fresh group is the highest. The firmness value of the Nanguo pears in the one cycle group have an average value of 309.00 g. The firmness value in the two cycles group (with an average value of 303.51 g and standard deviation of 60.51 g) is slightly lower than that in the one cycle group. The firmness value in the three cycles group is the lowest, with an average value of 273.92 g and a standard deviation of 84.10 g. Figure 6 shows that the values in the one cycle group and the two cycles group fluctuate around 300 g, while the firmness values in the three cycles group fluctuate around 250 g. It can be concluded that the fewer the cycles, the closer the firmness value is to that of the fresh group.

**Figure 7:**
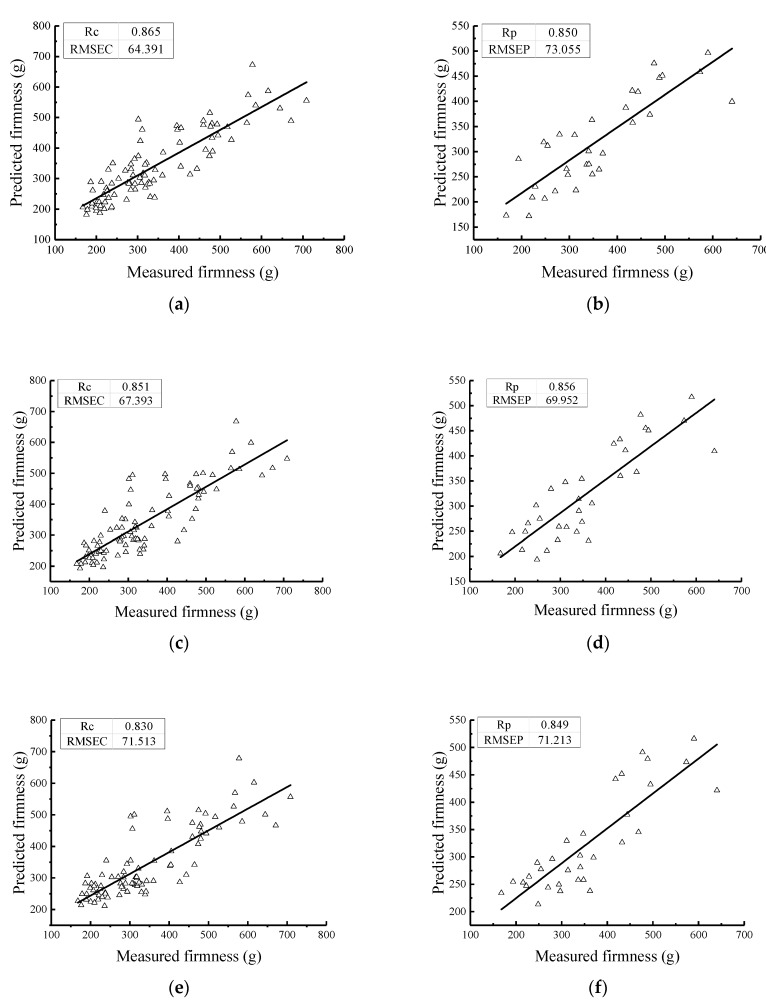

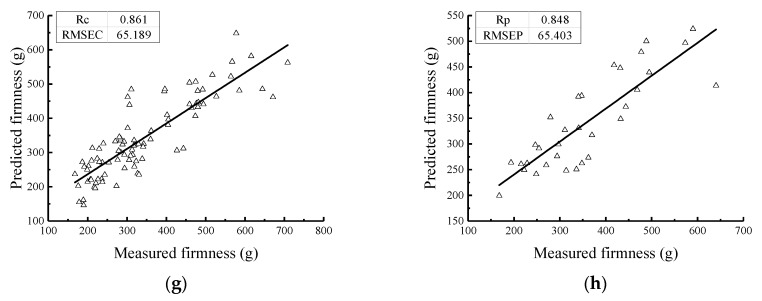
Prediction results of the firmness of the pears using the PLS method with different numbers of cycles: (**a**) the MSC calibration set; (**b**) the MSC validation set; (**c**) the S-G-MSC calibration set; (**d**) the S-G-MSC validation set; (**e**) the S-G-SNV calibration set; (**f**) the S-G-SNV validation set; (**g**) the SNV calibration set; and (**h**) the SNV validation set.

The PLS method was used to establish the firmness value model of the Nanguo pears. The results of the PLS models for the Nanguo pear firmness values from the calibration set and the validation set are shown in Figure 7; in these models, the Nanguo pears were treated with different numbers of cycles (fresh, one cycle, two cycles, and three cycles).

Figure 7 shows that the correlation coefficients of the calibration set and the validation set for the models established under different pretreatment methods are greater than 0.81, which shows that the model has a good performance and prediction ability. Comparing the RMSEC and the RMSEP in each processing method, the difference between them is the smallest after SNV pretreatment, which shows that this model is the best. This model is followed by the models in which the original spectrum was treated by S-G-MSC, S-G-SNV treatment, and finally, MSC. It can also be seen from Figure 7 that the measured values and the predicted values of the Nanguo pears are uniformly distributed on both sides of the fitting line in the SNV correction set. The firmness distribution of the Nanguo pears is more concentrated on both sides of the regression line than in the other three groups, which indicates that the predictive ability of this model is better and the accuracy is higher.

### 5.2. Firmness Prediction Model at Different Cooling Rates

In total, 88 Nanguo pear samples were used as calibration set samples. The samples were numbers 1 to 22 from the fresh group, numbers 31 to 52 from the 1 °C/min group, numbers 61 to 82 from the 3 °C/min group and numbers 91 to 112 from the 5 °C/min group. In total, 32 Nanguo pear samples, which were numbers 23 to 30 from the fresh group, numbers 53 to 60 from the 1 °C/min group, numbers 83 to 90 from the 3 °C/min group and numbers 113 to 120 from the 5 °C/min group, were used as validation set samples to carry out the modeling and comparative analysis. The experimental scheme is shown in Table 2.

**Figure 8:**
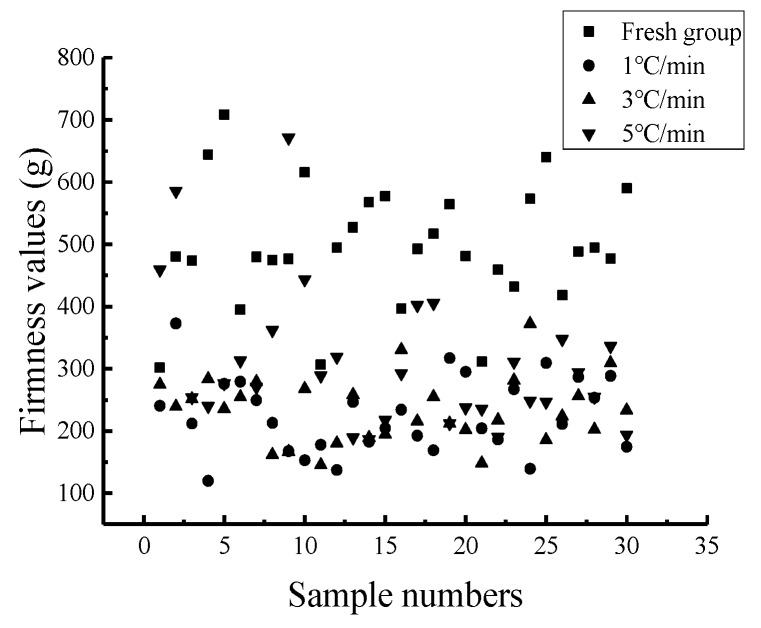
Scatter plot of the distribution of the firmness values of the Nanguo pears.

The statistical analysis results of the firmness values of the 120 Nanguo pears frozen at different cooling rates are shown in Table 8. The scatter plot of the firmness distribution of the Nanguo pears is shown in Figure 8.

From Figure 8, it can also be concluded that the firmness value of the fresh group is the largest, followed by the firmness value of the Nanguo pears subjected to a 5 °C/min cooling rate, with an average value of 309.00 g. The firmness of the Nanguo pears subjected to a cooling rate of 3 °C/min is slightly higher than that of the Nanguo pears subjected to a 1 °C/min cooling rate. The average values are 234.09 g and 225.25 g, respectively, and the standard deviations are approximately 60 g and 53 g, respectively. The faster the cooling rate, the better the firmness of Nanguo pear is maintained.

**Figure 9:**
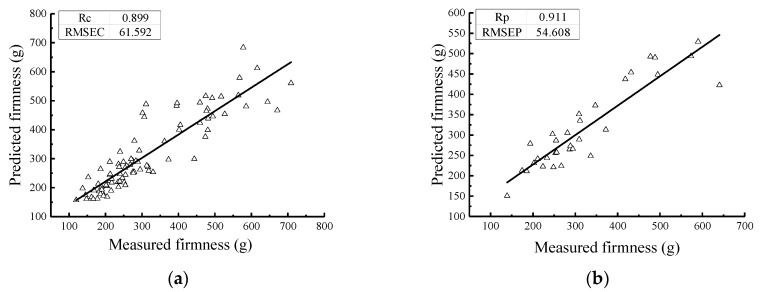

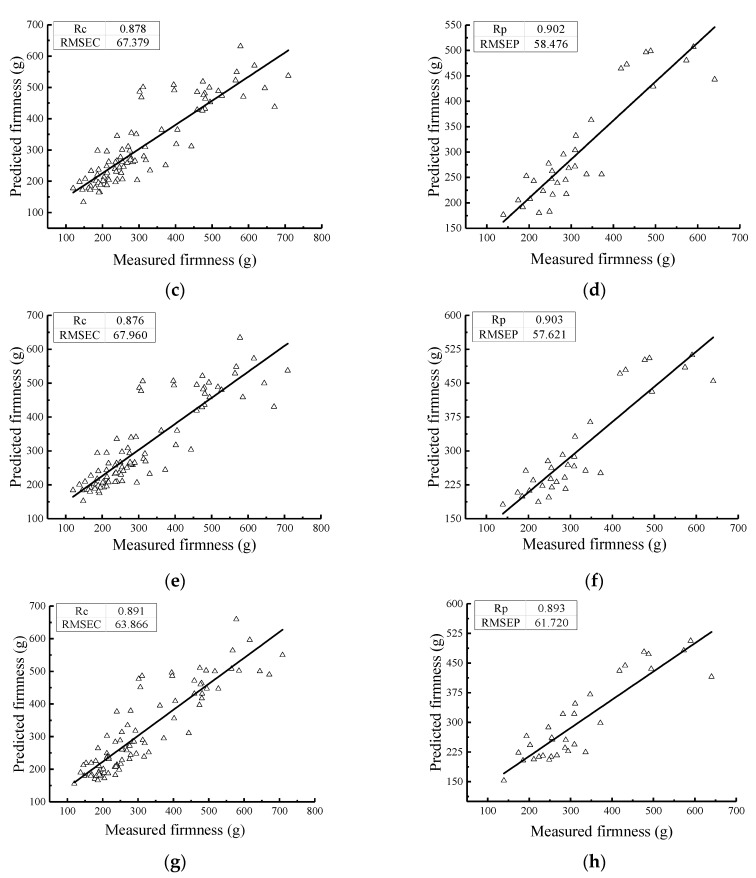
Prediction results of the firmness of pears using the PLS method with different cooling rates: (**a**) the MSC calibration set; (**b**) the MSC validation set; (**c**) the S-G-MSC calibration set; (**d**) the S-G-MSC validation set; (**e**) the S-G-SNV calibration set; (**f**) the S-G-SNV validation set; (**g**) the SNV calibration set; and (**h**) the SNV validation set.

The PLS method was used to establish the firmness value model of the Nanguo pears. The results of the PLS models for the Nanguo pear firmness values from the calibration set and the validation set are shown in Figure 9. The pears were treated with different cooling rates (fresh, 1 °C/min, 3 °C/min, and 5 °C/min). Figure 9 shows that the correlation coefficients of the calibration set and the validation set of the model with the different pretreatments are both greater than 0.85, which illustrates that the models both can predict unknown variables. The performance of the model with MSC pretreatment is the best; its correlation coefficient for the validation set is greater than 0.9, and the difference between the RMSEC and the RMSEP is small. Its predicted performance is excellent, and it can predict unknown variables well. The predicted and measured values of the samples are uniformly distributed on both sides of the regression line, which shows that the model has a better prediction accuracy, as shown in Figure 9.

### 5.3. Firmness Prediction Model at Different Holding Times

In total, 88 Nanguo pear samples were used as calibration set samples. The samples were numbers 1 to 22 of the fresh group, numbers 31 to 52 of the 30 min group, numbers 61 to 82 of the 45 min group and numbers 91 to 112 of the 60 min group. In total, 32 Nanguo pear samples, which were numbers 23 to 30 from the fresh group, numbers 53 to 60 from the 30 min group, numbers 83 to 90 from the 45 min group and numbers 113 to 120 from the 60 min group, were used as validation set samples to carry out the modeling and comparative analysis. The experimental scheme is shown in Table 3.

**Figure 10:**
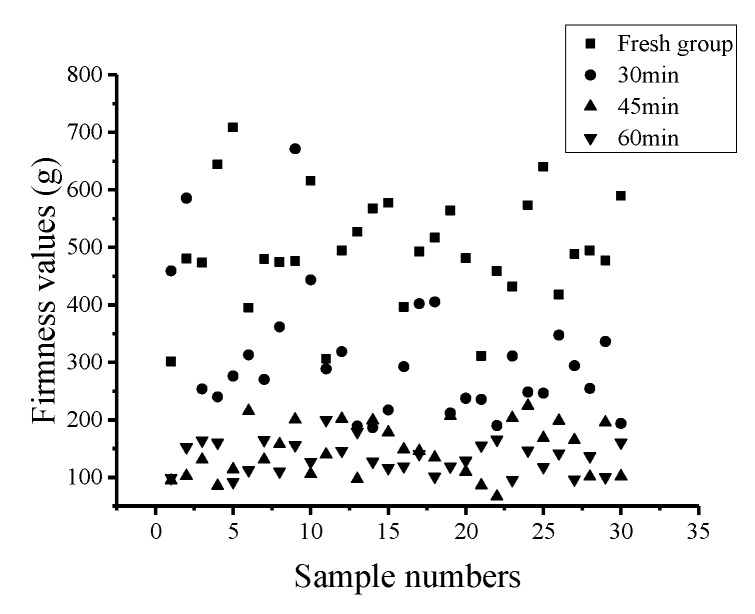
Scatter plot of the distribution of the firmness values of the Nanguo pears.

The statistical analysis results of the firmness values of the 120 Nanguo pears frozen at different holding times are shown in Table 9. The scatter plot of the firmness distribution is shown in Figure 10.

From Figure 10, it can be concluded that the firmness value in the fresh group is the largest: the average value is 495.34 g, the standard deviation is 96.11 g, and the firmness values of the individual samples are approximately 300 g. The firmness value in the Nanguo pears with a holding time of 30 min follows: the average value is 309.00 g, the standard deviation is 112.00 g, and the value of the individual samples is greater than 400 g. The firmness values in the Nanguo pears with holding times of 45 min and 60 min fluctuated around 140 g: the average values are 146.99 g and 134.51 g respectively, and the standard deviations are 45.84 g and 27.42 g, respectively. After a whole freezing/thawing process, it can be observed that the longer the holding time, the lower the firmness value.

**Figure 11:**
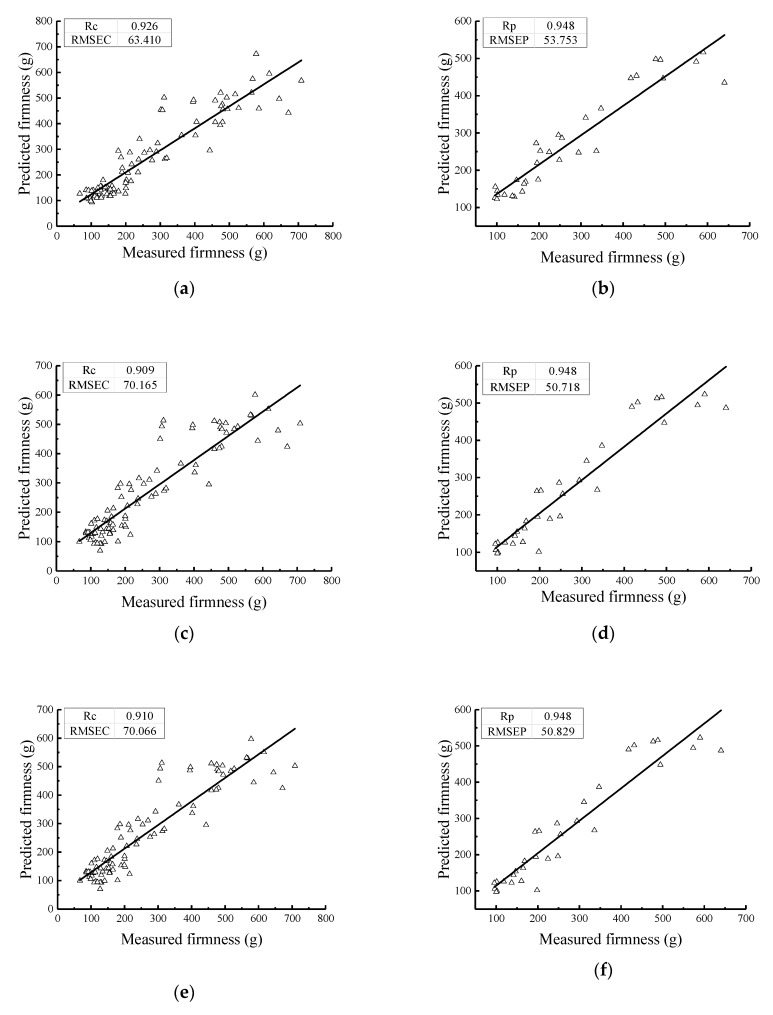

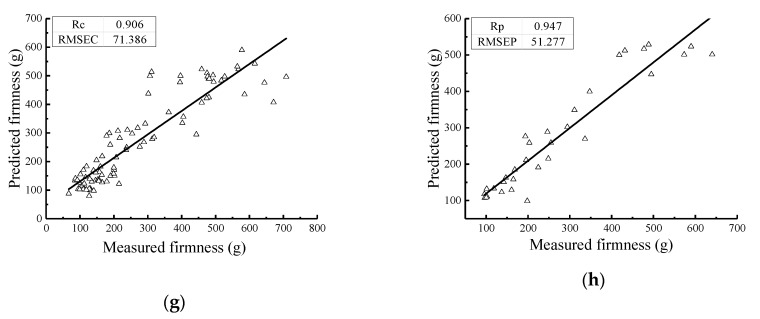
Prediction results of the firmness of the pears using the PLS method at different holding times: (**a**) the MSC calibration set; (**b**) the MSC validation set; (**c**) the S-G-MSC calibration set; (**d**) the S-G-MSC validation set; (**e**) the S-G-SNV calibration set; (**f**) the S-G-SNV validation set; (**g**) the SNV calibration set; and (**h**) the SNV validation set.

The PLS method was used to establish the firmness value model of the Nanguo pears. The results of the PLS model for the Nanguo pear firmness values from the calibration set and validation set are shown in Figure 11; in these models, the pears were frozen under different holding times (fresh, 30 min, 45 min, 60 min). Under freezing/thawing conditions with different holding times, the models of the average hyper spectrum of the Nanguo pears after four different pretreatments were established. The correlation coefficients of the calibration set are all greater than 0.90, and the correlation coefficients of the validation set are all above 0.94. As shown in Figure 11, the model after pretreatment has a better performance and a better prediction ability. The model after MSC pretreatment is the best. As shown in Figure 11, the measured values and predicted values under each processing method are uniformly distributed on both sides of the regression line, and this figure also shows precise representations of the models.

### 5.4. Firmness Prediction Model at Different Critical Temperatures

In total, 88 Nanguo pear samples were used as calibration set samples. The samples were numbers 1 to 22 from the fresh group, numbers 31 to 52 from the −10 °C group, numbers 61 to 82 from the −20 °C group and numbers 91 to 112 from the −30 °C group. In total, 32 Nanguo pear samples, which were numbers 23 to 30 from the fresh group, numbers 53 to 60 from the −10 °C group, numbers 83 to 90 from the −20 °C group and numbers 113 to 120 from the −30 °C group, were used as validation set samples to carry out the modeling and comparative analysis. The experimental scheme is shown in Table 4.

**Figure 12:**
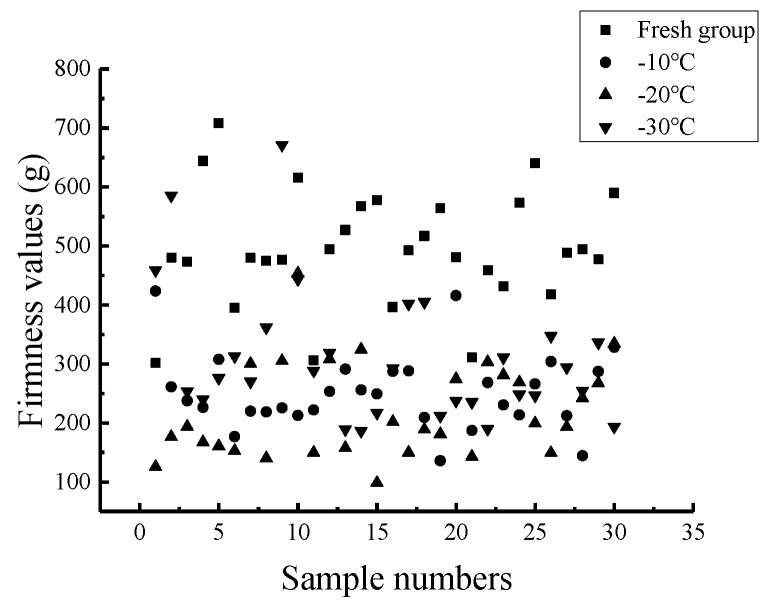
Scatter plot of the distribution of the firmness values of the Nanguo pears.

The statistical analysis results of the firmness values of the 120 Nanguo pears frozen at different critical temperatures are shown in Table 10. The scatter plot of the firmness distribution of the Nanguo pears is shown in Figure 12. From Figure 12, it can also be concluded that the firmness value of the Nanguo pear of the fresh group is the largest. The firmness value of the Nanguo pears, whose critical temperature is −30 °C, follows, with an average value of 309.00 g. The average firmness value of the Nanguo pears at the critical temperature of −10 ℃ is 252.23 g, and the standard deviation is 63.30 g. The average firmness value of the Nanguo pears at a critical temperature of −20 °C is approximately 220 g, and the standard deviation is approximately 80 g.

**Figure 13:**
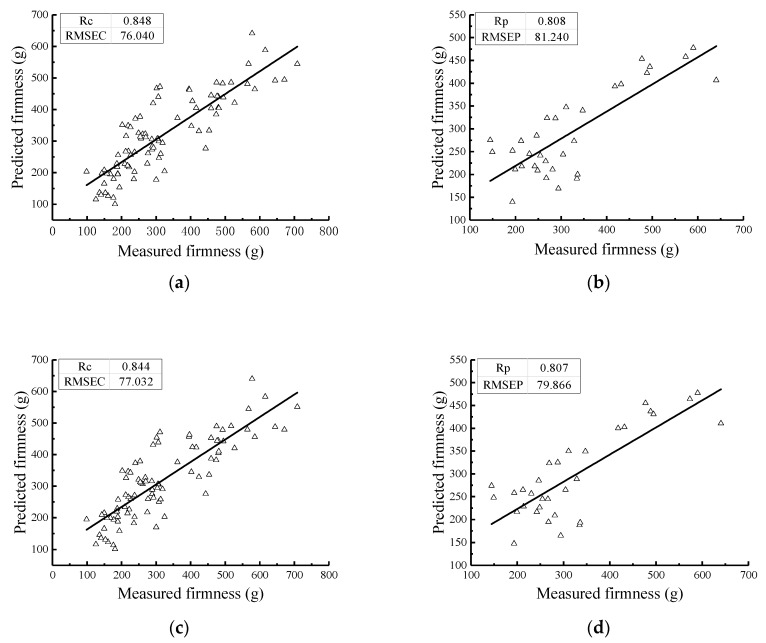

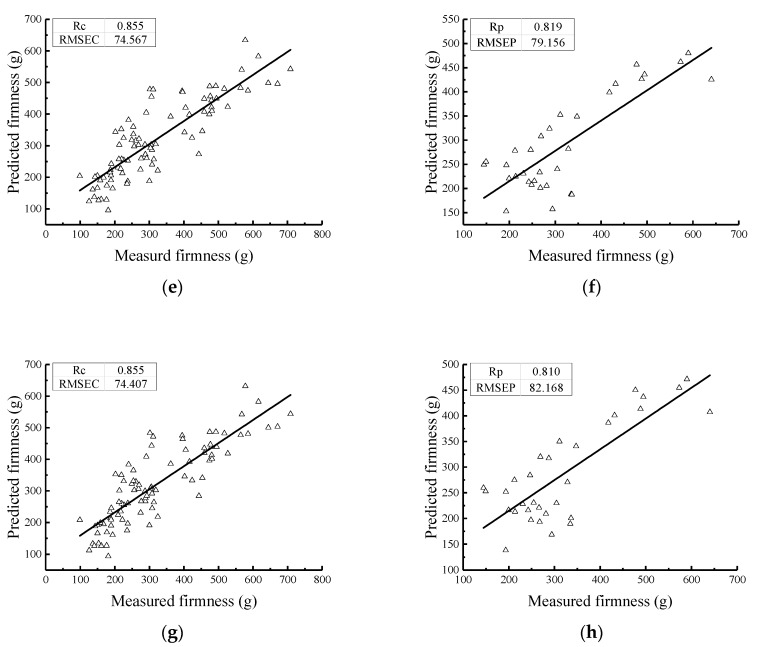
Prediction results of the firmness of the pears using the PLS method at different critical temperatures: (**a**) the MSC calibration set; (**b**) the MSC validation set; (**c**) the S-G-MSC calibration set; (**d**) the S-G-MSC validation set; (**e**) the S-G-SNV calibration set; (**f**) the S-G-SNV validation set; (**g**) the SNV calibration set; and (**h**) the SNV validation set.

The PLS method was used to establish the firmness value models of the Nanguo pears. The results of the PLS model of the Nanguo pear firmness value for the calibration set and the validation are shown in Figure 13. The pears were frozen at different critical temperatures (fresh, −10 °C, −20 °C, −30 °C).

Figure 13 shows that the models established by different pretreatments combined with the partial least squares algorithm roughly have the same performance and a good predictive ability. The model established by the S-G-SNV pretreatment combined with the PLS method is relatively optimal. From Figure 13, it can be found that the measured and predicted values are uniformly concentrated on both sides of the regression line, which shows that the model can predict the firmness value of unknown samples well. It can also be seen that the difference in the root mean square errors is small.

## 6. Conclusions

Hyperspectral imaging can reflect the change in the firmness of Nanguo pears well. In the waveband range of 950 nm-1400 nm, the firmness detection model of Nanguo pears based on PLS was established. The spectral reflectivity of Nanguo pears is diverse under different freezing/thawing conditions, but the total change trend in the spectral reflectivity is the same, and the reflectivity of fresh pears is the highest. In other words, the higher the cooling rate is, the shorter the holding time, the higher the terminal temperature, the fewer the number of cycles and the higher the reflectivity of Nanguo pears. The following conclusions were obtained after applying four different freezing/thawing schemes:

(1) By comparing the RMSEC and the RMSEP under each processing mode for different numbers of cycles, it can be observed that the difference between them is the smallest after the SNV pretreatment, which indicates that the model is the best.
(2) The model established after MSC pretreatment has the best performance at different cooling rates, and the difference between the RMSEC and the RMSEP is relatively small, so it can predict unknown variables well.
(3) Under the freezing processing conditions at different holding times, the correlation coefficients of the validation set are all above 0.94, which indicates that the predictive ability of the model after pretreatment is greatly improved. The model established after MSC pretreatment is the best.
(4) Under the freezing conditions at different critical temperatures, the model established after S-G-SNV pretreatment combined with PLS is relatively optimal.

## Figures and Tables

**Table 1:** Standard experimental scheme.

Group	Starting Temperature (°C)	Cooling Rate (°C/min)	Transition Temperature (°C)	Critical Temperature (°C)	Holding Time (min)	Thawing Rate (°C/min)	Final Thawing Final Temperature (°C)
Standard group	22	5	−3	−30	30	10	25

**Table 2:** Experimental scheme at different cooling rates.

Group	Starting Temperature (°C)	Cooling Rate (°C/min)	Transition Temperature (°C)	Critical Temperature (°C)	Holding Time (min)	Thawing Rate (°C/min)	Final Thawing Temperature (°C)
Second group	22	1	−3	−30	30	10	25
Third group	22	3	−3	−30	30	10	25
Fourth group	22	5	−3	−30	30	10	25

**Table 3:** Experimental scheme at different holding times.

Group	Starting Temperature (°C)	Cooling Rate (°C/min)	Transition Temperature (°C)	Critical Temperature (°C)	Holding Time (min)	Thawing Rate (°C/min)	Final Thawing Temperature (°C)
Second group	22	5	−3	−30	30	10	25
Third group	22	5	−3	−30	45	10	25
Fourth group	22	5	−3	−30	60	10	25

**Table 4:** Experimental scheme at different critical temperatures.

Group	Starting Temperature (°C)	Cooling Rate (°C/min)	Transition Temperature (°C)	Critical Temperature (°C)	Holding Time (min)	Thawing Rate (°C/min)	Final Thawing Temperature (°C)
Second group	22	5	−3	−10	30	10	25
Third group	22	5	−3	−-20	30	10	25
Fourth group	22	5	−3	−30	30	10	25

**Table 5:** The extraction values of the characteristic wavelengths for different numbers of cycles.

Different Freezing Condition	Pretreatment Method	Characteristic Wavelengths (nm)
Different numbers of cycles	MSC	950, 953, 956, 962, 977, 986, 989, 1052, 1118, 1124, 1181, 1301, 1304, 1313, 1316, 1319, 1349
	S-G-MSC	974, 977, 986, 1028, 1031, 1046, 1088, 1118, 1145, 1247, 1301, 1304, 1316, 1349
	SNV	956, 974, 986, 989, 992, 1028, 1052, 1085, 1247, 1322, 1355
	S-G-SNV	974, 977, 989, 1031, 1049, 1055, 1250, 1298, 1301, 1304, 1316, 1349

**Table 6:** The firmness values of the Nanguo pears.

**Sample**	**Fresh (g)**	**1 °C/min(g)**	**3 °C/min(g)**	**45 min (g)**	**60 min (g)**
1	301.73	240.37	274.67	94.70	98.99
2	480.37	372.79	239.19	102.30	152.60
3	473.75	211.73	251.26	130.68	164.21
4	644.40	119.72	283.22	85.48	160.30
5	708.61	275.59	235.60	113.98	91.96
6	395.01	279.11	254.44	215.33	112.59
7	479.75	249.48	279.04	130.68	165.23
8	474.77	212.94	161.75	157.65	110.29
9	476.62	167.52	166.41	200.47	155.98
10	615.90	152.72	267.71	105.91	127.16
11	306.46	177.60	145.28	139.77	199.94
12	494.51	137.24	180.20	201.98	146.35
13	527.09	246.40	257.89	97.13	178.99
14	567.54	182.78	188.98	199.15	127.37
15	577.78	204.41	194.66	178.48	116.55
16	396.57	234.23	330.18	148.76	119.33
17	492.54	192.39	215.40	145.79	140.53
18	517.15	168.89	254.83	134.46	101.21
19	564.21	317.04	212.08	206.64	118.93
20	481.30	294.96	201.63	109.62	128.90
21	311.16	203.97	147.92	86.33	155.31
22	459.03	186.11	217.32	66.78	165.41
23	431.67	267.11	281.50	203.18	95.60
24	573.34	139.05	372.12	224.43	146.86
25	640.39	309.31	185.27	168.36	118.33
26	418.07	211.27	223.64	198.27	141.90
27	488.44	286.67	255.99	164.88	96.60
28	494.72	253.30	202.37	101.14	136.98
29	477.32	288.38	309.00	195.51	100.31
30	590.02	174.41	233.28	101.81	160.62
**Sample**	**One cycle (g)**	**Two cycles (g)**	**Three cycles (g)**	**−10 °C (g)**	**−20 °C (g)**
1	459.20	321.72	202.16	423.77	125.75
2	585.57	342.49	191.44	261.11	176.35
3	253.99	224.43	330.67	237.61	193.87
4	239.96	226.33	229.27	226.33	167.80
5	276.36	293.87	464.44	307.91	160.87
6	313.20	283.77	338.99	177.12	153.04
7	270.31	326.96	426.97	219.91	300.27
8	361.81	318.18	176.21	219.03	140.67
9	671.25	243.59	207.00	225.73	305.39
10	443.44	284.19	208.81	213.15	453.36
11	288.78	219.26	341.07	222.02	149.82
12	318.76	314.68	278.86	253.72	308.12
13	189.23	317.32	305.41	291.25	157.86
14	186.55	283.45	237.57	256.31	324.48
15	217.20	309.17	178.41	249.29	98.71
16	292.53	280.81	323.46	287.64	202.07
17	401.94	359.93	273.23	288.50	149.84
18	405.16	221.12	199.17	209.56	189.10
19	212.01	200.17	210.13	136.24	181.17
20	237.50	404.44	167.71	415.98	274.64
21	235.65	293.43	229.67	187.50	142.89
22	190.02	301.77	329.42	268.69	303.51
23	311.11	370.04	167.89	230.66	281.41
24	248.51	222.48	340.35	213.86	269.20
25	246.51	348.02	362.23	266.28	199.60
26	347.40	432.48	228.60	304.37	149.31
27	294.11	279.44	313.87	212.45	193.19
28	254.58	296.95	468.56	144.58	242.38
29	336.25	340.98	270.33	287.59	267.67
30	193.61	443.67	215.53	328.58	334.82

**Table 7:** Firmness value of Nanguo pears.

Index	Samples	Average Value	Standard Deviation	Minimum Value	Maximum Value
Firmness (g)	Fresh	495.34	96.11	301.73	708.61
	One cycle	309.00	112.00	186.55	671.25
	Two cycles	303.51	60.51	200.17	443.67
	Three cycles	273.92	84.10	167.71	468.56

**Table 8:** Firmness values of the Nanguo pears.

Index	Samples	Average Value	Standard Deviation	Minimum Value	Maximum Value
Firmness (g)	Fresh	495.34	96.11	301.73	708.61
	1 °C/min	225.25	59.99	119.72	382.79
	3 °C/min	234.09	52.55	145.28	330.18
	5 °C/min	309.00	112.00	186.55	671.25

**Table 9:** Firmness values of the Nanguo pears.

Index	Samples	Average Value	Standard Deviation	Minimum Value	Maximum Value
Firmness (g)	Fresh	495.34	96.11	301.73	708.61
	30 min	309.00	112.00	186.55	671.25
	45 min	146.99	45.84	85.48	224.43
	60 min	134.51	27.42	91.96	199.94

**Table 10:** Firmness values of the Nanguo pears.

Index	Samples	Average Value	Standard Deviation	Minimum Value	Maximum Value
Firmness (g)	Fresh	495.34	96.11	301.73	708.61
	−10 °C	252.23	63.30	136.24	423.77
	−20 °C	219.90	79.73	98.71	453.36
	−30 °C	309.00	112.00	186.55	671.25

## References

[1] Kobayashi R., Suzuki T. (2019). Effect of supercooling accompanying the freezing process on ice crystals and the quality of frozen strawberry tissue. Int. J. Refrig..

[2] Xu Y., Hua T.C., Sun D.W., Zhou G.Y. (2008). Experimental study and analysis of mechanical properties of frozen rabbit aorta by fracture mechanics approach. J. Biomech..

[3] Ali U., Kanwar S., Yadav K., Basu S., Mazumder K. (2019). Effect of arabinoxylan and β-glucan stearic acid ester coatings on post-harvest quality of apple (Royal Delicious). Carbohyd. Polym..

[4] Wang Y., Lv Y., Liu H., Wei Y., Zhang J., An D., Wu J. (2018). Identification of maize haploid kernels based on hyperspectral imaging technology. Comput. Electron. Agr..

[5] Veraverbeke S., Dennison P., Gitas I., Hulley G., Kalashnikova O., Katagis T., Kuai L., Meng R., Roberts D., Stavros N. (2018). Hyperspectral remote sensing of fire: State-of-the-art and future perspectives. Remote Sens. Environ..

[6] Cleemput E.V., Vanierschot L., Fernández-Castilla B., Honnay O., Somers B. (2018). The functional characterization of grass- and shrubland ecosystems using hyperspectral remote sensing: trends, accuracy and moderating variables. Remote Sens. Environ..

[7] Zhang H.Y., Ren X.X., Zhou Y., Wu Y.P., He L., Heng Y.R., Feng W., Wang C.Y. (2018). Remotely assessing photosynthetic nitrogen use efficiency with in situ hyperspectral remote sensing in winter wheat. Eur. J. Agron..

[8] Sandasi M., Chen W.Y., Vermaak I., Viljoen A. (2018). Non-destructive quality assessment of herbal tea blends using hyperspectral imaging. Phytochem. Lett..

[9] Suktanarak S., Teerachaichayut S. (2017). Non-destructive quality assessment of hens’ eggs using hyperspectral images. J. Food Eng..

[10] Siripatrawan U., Makino Y. (2018). Simultaneous assessment of various quality attributes and shelf life of packaged bratwurst using hyperspectral imaging. Meat Sci..

[11] Bowling M.B., Vote D.J., Belk K.E., Scanga J.A., Tatum J.D., Smith G.C. (2009). Using Reflectance Spectroscopy to Predict Beef Tenderness. Meat Sci..

[12] Siedliska A., Baranowski P., Zubik M., Mazurek W., Sosnowska B. (2018). Detection of fungal infections in strawberry fruit by VNIR/SWIR hyperspectral imaging. Postharvest Biol. Tec..

[13] ElMasry G., Wang N., Vigneault C., Qiao J., ElSayed A. (2008). Early detection of apple bruises on different background colors using hyperspectral imaging. LWT Food Sci. Technol..

[14] Ariana D.P., Lu R., Guyer D.E. (2006). Near-infrared hyperspectral reflectance imaging for detection of bruises on pickling cucumbers. Comput. Electron. Agr..

[15] Lee W.H., Kim M.S., Lee H., Delwiche S.R., Bae H., Kim D.Y., Cho B.K. (2014). Hyperspectral near-infrared imaging for the detection of physical damages of pear. J. Food Eng..

[16] Fan S., Huang W., Guo Z., Zhang B., Zhao C. (2015). Prediction of Soluble Solids Content and Firmness of Pears Using Hyperspectral Reflectance Imaging. Food Anal. Method..

[17] Li B., Hou B., Zhang D., Zhou Y., Zhao M., Hong R., Huang Y. (2016). Pears characteristics (soluble solids content and firmness prediction, varieties) testing methods based on visible-near infrared hyperspectral imaging. Optik.

[18] Yu K.Q., Zhao Y.R., Liu Z.Y., Li X.L., Liu F., He Y. (2014). Application of Visible and Near-Infrared Hyperspectral Imaging for Detection of Defective Features in Loquat. Food Bioprocess Tech..

[19] Cai Z.Y., Wu L.G., Wang J., Pan Y., Ma J.R., Li Z.Y. (2017). Non-destructive determination of moisture composition in Ningxia wine grapes based on visible near-infrared hyperspectral imaging technique. Sci. Technol. Food Ind..

[20] Nicolaï B.M., Lötze E., Peirs A., Scheerlinck N., Theron K.I. (2006). Non-destructive Measurement of Bitter Pit in Apple Fruit Using NIR Hyperspectral Imaging. Postharvest Biol. Tec..

[21] Wei Y., Wu F., Xu J., Sha J., Zhao Z., He Y., Li X. (2019). Visual detection of the moisture content of tea leaves with hyperspectral imaging technology. J. Food Eng..

[22] Tung K.C., Tsai C.Y., Hsu H.C., Chang Y.H., Chang C.H., Chen S. (2018). Evaluation of Water Potentials of Leafy Vegetables Using Hyperspectral Imaging. IFAC-PapersOnLine.

[23] Huang M., Wang Q., Zhang M., Zhu Q. (2014). Prediction of color and moisture content for vegetable soybean during drying using hyperspectral imaging technology. J. Food Eng..

[24] Shen R.L. (1994). Temperature and Humidity Regulated Method of Constant Temperature and Humidity Box. Environ. Technol..

[25] Zhang B.H., Li J.B., Fan S.X., Huang W.Q., Zhang C., Wang Q.Y., Xiao G.D. (2014). Principle and Application of Hyperspectral Imaging Technology in Non-destructive Testing of Fruit and Vegetable Quality and Safety. Spectrosc. Spect. Anal..

[26] Ma B.X. (2009). Methodology for Rapid and Nondestructive Detection of Fruit Quality Based on Image Processing and Spectral Analysis Technologies. Ph.D. Thesis.

[27] Zhu F.L. (2014). Rapid and non-destructive detection of marine fish quality based on spectroscopy and hyperspectral imaging technique. Ph.D. Thesis.

[28] Helland I.S., Næs T., Isaksson T. (1995). Related versions of the multiplicative scatter correction method for preprocessing spectroscopic data. Chemometr. Intell. Lab..

[29] Li D. (2015). Non-destructive Detection of Quality in Lingwu Jujube Based on Hyperspectral Imaging Technology. Ph.D. Thesis.

[30] Savitzky A., Golay M.J.E. (1964). Smoothing and Differentiation of Data by Simplified Least Squares Procedures. Anal. Chem..

[31] Burger J., Gowen A. (2011). Data handling in hyperspectral image analysis. Chemometr. Intell. Lab..

[32] Wu D., Sun D.W. (2013). Potential of time series-hyperspectral imaging (TS-HSI) for non-invasive determination of microbial spoilage of salmon flesh. Talanta.

[33] Li H., Liang Y., Xu Q., Cao D. (2009). Key wavelengths screening using competitive adaptive reweighted sampling method for multivariate calibration. Anal. Chim. Acta.

[34] Xu D., Wang H., Ji H., Zhang X., Wang Y., Zhang Z., Zheng H. (2018). Hyperspectral Imaging for Evaluating Impact Damage to Mango According to Changes in Quality Attributes. Sensors.

